# Risk factors for construct/implant related complications following primary posterior hemivertebra resection: Study on 116 cases with more than 2 years’ follow-up in one medical center

**DOI:** 10.1186/s12891-016-1229-y

**Published:** 2016-09-02

**Authors:** Jianwei Guo, Jianguo Zhang, Shengru Wang, Hai Wang, Yanbin Zhang, Yang Yang, Xinyu Yang, Lijuan Zhao

**Affiliations:** 1 Department of Orthopedics, The Affiliated Hospital of Qingdao University, Qingdao, 266003 People’s Republic of China; 2 Department of Orthopedics, Peking Union Medical College Hospital, 1 Shuai Fu Yuan, Beijing, 100730 People’s Republic of China

**Keywords:** Congenital scoliosis, Posterior hemivertebra resection, Complication, Risk factor

## Abstract

**Background:**

Posterior hemivertebra resection has become a safe and effective procedure for congenital scoliosis due to hemivertebra. However, there are still complications following primary posterior hemivertebra resection in recent reports. No risk factors associated with construct/implant related complications were identified so far. The purpose of this study is to analyze complications following primary posterior hemivertebra resection and to investigate the possible risk factors associated with construct/implant related complications in congenital scoliosis cases due to hemivertebra.

**Methods:**

One hundred and sixteen congenital scoliosis cases with hemivertebra (male: female = 62:54), who underwent primary posterior hemivertebra resection from January 2003 to January 2012 in our medical center, were retrospectively evaluated in this study. Medical records were reviewed and long cassette standing spinal radiographs were measured before surgery, after surgery and at the final follow-up. Complications, including construct/implant related complications and non-construct/implant related ones, were recorded by chart review. Potential risk factors, including sex, age, segmental and main scoliosis and their correction rates, usage of cage (yes or not), fusion levels (bisegmental fusion or not), location of hemivertebra and contralateral bar/rib synostosis (with or without), were also collected.

**Results:**

The mean age of initial surgery was 9.8 years old (range, 2–19 years), and the average follow-up was 67 months (range, 24–133 months). The segmental scoliosis was corrected from 34.7° ± 11.9° to 7.1° ± 5.6° post-operatively, and 9.5° ± 7.0° at the latest follow-up, with a correction rate of 79.5 %. The total main scoliosis was corrected from 43.0° ±15.6° to post-operative 11.4° ± 6.8°, and 13.9° ± 7.5° at the latest follow-up, with a mean correction rate of 73.5 %. Complications occurred in 11 out of 116 cases (9.5 %), including 7 (63.6 %) construct/implant related complications (two pedicle misplacement, one rod breakage and four implant removals due to screw dislodgement with the growth) and 4 (36.4 %) non-construct/implant related ones (one proximal adjacent kyphosis, one progressive kyphosis, and two wound non-union). Younger age (≤5 years old), lumbar hemivertebra resection, or bisegmental fusion may contribute to a higher prevalence of construct/implant related complications than other cases, although the number of cases was too small to perform statistical analysis.

**Conclusions:**

The occurrence of construct/implant related complications in patients with hemivertebra resection is most likely multifactorial. Cases with younger age, bisegmental fusion, or lumbar hemivertebra may increase the risk of construct/implant related complications. Measures, such as careful preoperative evaluation and surgical plan with CT scan, sophisticated operation during surgery, usage of cage or cross-links to improve postoperative instant stability, protection in brace and regular follow-up postoperatively, should be taken to reduce construct/implant related complication rate.

## Background

Hemivertebrae is the most frequent cause of congenital scoliosis [1]. Except for the incarcerated, unsegmented or balanced ones, most hemivertebra have growth potential similar to a normal vertebrae and create a wedge-shaped deformity during spinal growing period. In general, the curve progresses 1–3.5° per year and develops a severe one at the skeletal maturity, especially for a lower thoracic or thoracolumbar hemivertebra [2]. Thus, earlier surgical intervention is suggested in most cases [3–18].

Since hemivertebra resection was first described by Royle in 1928 [19], several surgical procedures have been proposed to deal with hemivertebra [4, 5, 8, 12, 18, 20]. Compared with one-stage or two-stage anterior-posterior hemivertebra resection, the advantages of posterior hemivertebra resection, including good correction results in the coronal and sagittal plane, less invasive, lower complication rate and shorter postoperative recovery period, make it more suitable for young children to deal with this kind of deformity [6]. Although posterior hemivertebra resection have been proven to be a safe and effective procedure for congenital scoliosis due to hemivertebra, there are still complications following posterior hemivertebra resection. The reported complication rate is 0–28.5 % and most of the complications are associated with construct/implant failure [8, 10, 12–14, 16–18, 21]. As far as we know, there is still no reports about risk factors associated with construct/implant related complications. The purpose of this study is to analyze complications following primary posterior hemivertebra resection and to investigate the possible risk factors associated with the occurrence of construct/implant related complications.

## Methods

### Patients

After approval of the Ethics Committee of Peking Union Medical College Hospital, patients with posterior hemivertebra resection were retrospectively reviewed in our hospital. Inclusion of this study consisted of 1) age <20 years old at the initial surgery; 2) all performed by posterior hemivertebra resection with transpedicular instrumentation; 3) at least 2 years’ follow-up; 4) simple type of congenital deformity, including one or two hemivertebra at the same spinal region; 5) complete radiographic follow-up data. Patients with age more than 20 years old, previous spinal surgery, complex congenital spinal deformity, or syndromic scoliosis were excluded from this study.

From January 2003 to January 2012, 116 patients matched these criteria. These patients included 54 females and 62 males, with a mean age of 9.8 years at the initial surgery (range 2–19 years). 115 patients had only one hemivertebra resection, and only one patients had two hemivertebra resection at the same spinal region (L3 and L4/5 hemivertebra at lumbar region).

Clinical data (age & sex), radiographic data (location of the hemivertebrae, contralateral bar/rib synostosis or not, Cobb angle of main and segmental scoliosis, segmental kyphosis/lordosis), and the follow-up time were obtained through chart review. Cobb angles of main and segmental scoliosis were measured as Ruf’s description before surgery, after surgery and at last follow-up [8]. All the measurements were performed by two experienced surgeons independently.

### Surgical procedures

All the patients were treated with posterior hemivertebra resection using transpedicular implants by the correspondence author. Intraoperative monitoring with combination of sensory evoked potential (SEP) and motor evoked potential (MEP) were performed during the operation. The patient was positioned prone on a radiolucent operation table after general anesthesia. For cases with only one hemivertebra, different fusion strategies were used in this study: bisegmental fusion for lumbar hemivertebra, the upper two and lower one or two segmental fusion for thoracolumbar hemivertebra, and the upper two and lower two segmental fusions for thoracic hemivertebra.

After exposure of the hemivertebra and vertebra to be fused, pedicle screws were carefully placed under the guide of fluoroscopy. Pedicle instrumentation, including CDH (Medtronic, Titanium, 39cases), ISOLA (Depuy, Steel, 6 cases), Legacy (Medtronic, Titanium, 8 cases), Moss SI (Depuy, Titanium, 17cases), Moss Miami (Medtronic, Titanium, 12cases), Sino (WEGO, Titanium, 2cases), TSRH (Medtronic, Titanium, 2cases), USS (AO, Titanium, 13cases), XIA 4.5 (Stryker, Titanium, 17cases), were used in our operation. There were 111 cases with all pedicle screws and 5 cases with hybrid instrumentation of pedicle screws and hooks. Titanium mesh cages were used in 15 cases. For young cases, pedicle screw with a diameter of 3.5 mm to 5 mm and length of 20 to 30 mm were used.

The posterior elements of the hemivertebra, including the lamina, upper and lower facets and the transverse process were removed to expose the pedicle and the nerve roots above and below. The vertebra body, the upper and lower discs, cartilage and annulus fibrosus were removed till the bleeding bone. The opposite disc and cartilage should be resected as far as possible. Before compression, vertebral barrier structure to compression should be removed. In patients with contralateral bar and rib synostosis, the bar should be cut and the synostosed rib heads should be removed before compression. The osteotomy gap was closed by compression plier gradually and carefully. In patients with large hemivertebra or with obvious kyphosis, a mesh cage with allogenic bone (12 mm or 14 mm for young cases) was applied to reconstruct the anterior column of the spine. After compression, examination was needed to make sure that the exiting nerve roots and the dura were not impinged.

All patients were mobilized after the drainage tube was removed, and plastic braces were recommended to wear for at least 3 months. All the patients were followed at 3, 6, and 12 months after the surgery and then at a year interval. All the complications, including construct/implant related complications (e.g. pedicle fracture, screws out, the breakage of the rods & misplaced pedicle screw) and non- construct/implant related complications (e.g. infections, poor wound healing, cerebrospinal fluid leakage & neurological complications), were recorded in perioperative period and follow-up.

### Statistical analysis

All the continuous variables were described by means and proportions. All potential risk factors, including sex, age (≤5 years/old or >5 years/old), fusion levels (bisegmental fusion or not), location of the resected hemivertebra (lumbar or not), contralateral bar/rib synostosis (with or without), pre-operative Cobb angles of segmental and main scoliosis, and their correction rates, were collected. In order to eliminate the bias of age, all the cases were stratified into 3 groups, that is, group A (≤5 years old), group B (6 ~ 10 years old) and group C (>10 years old) for the analysis.

## Results

### General characteristics

The mean age was 9.8 years old (range: 2–19 years old). Their average follow-up period was 67 months (range, 24–133months). 117 hemivertebra resections were performed in this study (one case had two hemivertebra resection). The mean fusion level was 5 segments (range: 2–11 segments). The segmental scoliosis was corrected from 34.7° ± 11.9° to 7.1° ± 5.6° post-operatively, and 9.5° ± 7.0° at the latest follow-up, with a correction rate of 79.5 %. The total main scoliosis was corrected from 43.0° ±15.6° to post-operative 11.4° ± 6.8°, and 13.9° ± 7.5° at the latest follow-up, with a correction rate of 73.5 %. The segmental kyphosis/lordosis was corrected from 25.2° ± 20.4° to post-operative 6.2° ± 6.1°, and 6.9° ± 6.6° at the latest follow-up, with a correction rate of 75.4 %. The compensatory cranial curve were respectively 18.6° ± 9.5° before surgery, 10.9° ± 7.1°after surgery, and 10.4° ± 6.5° at the last follow-up, with a correction rate of 41.4 %. The compensatory caudal curve were respectively 20.2° ± 11.6° before surgery, 7.4° ± 5.0° after surgery, and 8.4° ± 5.3° at the latest follow-up, with the correction rate of 63.4 %.

In 116 cases, 36 cases were performed with bisegmental fusion (one vertebrae above and below the hemivertebra), which accounted for 31.0 %. The segmental scoliosis was corrected from 30.0° ± 8.0° to 4.8° ± 3.7° post-operatively, and 7.4° ± 4.8° at the latest follow-up, with a correction rate of 84 %. The main scoliosis was corrected from 34.9° ±8.6° to post-operative 8.9° ± 5.1°, and 11.6° ± 5.8° at the latest follow-up, with a correction rate of 74.5 %. The segmental kyphosis/lordosis was corrected from 16.2° ± 10.1° to post-operative 7.0° ± 5.3°, and 6.8° ± 5.4° at the latest follow-up, with a correction rate of 56.8 %. The spontaneous correction rate of the compensatory cranial and caudal curve was 42.7 and 48.1 %.

### Complications

Of 116 patients, there were 11 (9.5 %) patients who suffered from complications during the perioperative and follow-up periods, including 7 (6.0 %) construct/implant related ones and 4 (3.4 %) non- construct/implant related ones (Tables 1 and 2). There were 2 neurologic complications (Asia D) occurred associated with construct/implant related complications, one of which had left thigh pain, numbness and powerless, and the other had right calf and foot pain and numbness. And the symptom completely relieved after the revision surgery. No permanent neurologic complications were observed in this study.

**Table 1:** Summary of clinical data in complication cases

No.	Initial age (years/old)	Sex	Hemivertebra location	Fusion levels	Time of complication occurrence	Complications	Treatment
1	3	F	L2	L1-L3	4 years postoperative	screw migration	Remove implants
2	3	F	L2	L1-L3	7 years postoperative	screw migration	Remove implants
3	3	F	L4	L3-L5	6 months postoperative	rod break without migration	Conservative observation
4	5	F	L2	L1-L3	3 days postoperative	screw misplacement	Revision with hooks
5	17	F	L4	L2-S1	2 days postoperative	screw misplacement	Remove misplaced screws
6	7	M	L3/4	L3-L4	7 years postoperative	pedicle elongation and screw migration	Remove implants
7	2	F	L5/6	L5-L6	3 years postoperative	pedicle elongation and screw migration	Remove implants
8	15	M	T11/12	T8-L2	4 years postoperative	Proximal adjacent kyphosis	Extended fused segments
9	3	M	T11	T10-T12	7 years postoperative	Progressive kyphosis	Extended fused segments
10	15	F	T12/L1	T12-L2	8 days postoperative	Poor wound healing	Debridement
11	11	M	L1	T11-L2	6 days postoperative	Poor wound healing	Debridement

**Table 2:** Summary of radiograph data in complication cases

No.	Coronal segmental curve (°)			Total main curve (°)			Sagittal kyphosis (+)/lordosis (−)						
							Measured value (°)				Distance to norm (°)		
	Pre-op.	Postop.	Latest follow-up	Pre-op.	Postop.	Latest follow-up	Pre-op.	Postop.	Latest follow-up	Norm (°)	Pre-op.	Postop.	Latest follow-up
1	31	0	0	40	5	0	−5	−4	−6	−11	6	7	5
2	37	3	12	28	4	15	20	−4	−2	−11	31	7	9
3	30	12	20	42	14	23	−25	−17	−22	−20	−5	3	−2
4	21	9	0	37	15	0	14	−3	−6	−4	18	1	−2
5	34	16	15	41	21	17	14	−11	−11	−33	47	22	22
6	26	2	4	23	5	4	4	0	−7	−13	17	13	6
7	15	2	2	22	12	4	3	1	−6	−20	23	21	14
8	64	30	32	66	40	43	88	10	14	2.5	85.5	7.5	11.5
9	47	10	6	42	16	7	22	14	10	5.5	16.5	8.5	4.5
10	35	6	8	47	16	10	39	9	10	3.5	35.5	5.5	6.5
11	43	3	3	46	5	5	2	7	−3	−3	5	10	0

**Fig. 1:**
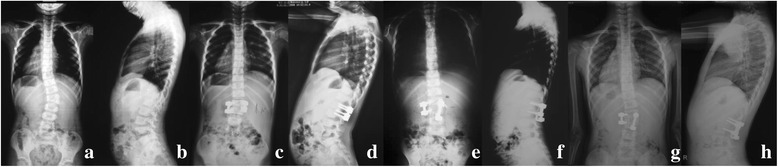
A 5-year-old girl with congenital scoliosis. Pre-operative radiographs (**a**, **b**) showed semi-segmented hemivertebra of L2. She complained of right leg pain on the third post-operational day. Post-operational radiographs (**c**, **d**) showed right L3 pedicle screw in wrong place. Revision surgery was performed with hooks, and his leg pain disappeared after the revision surgery (**e**, **f**). And it maintained well in 7 years follow-up (**g**, **h**)

**Fig. 2:**
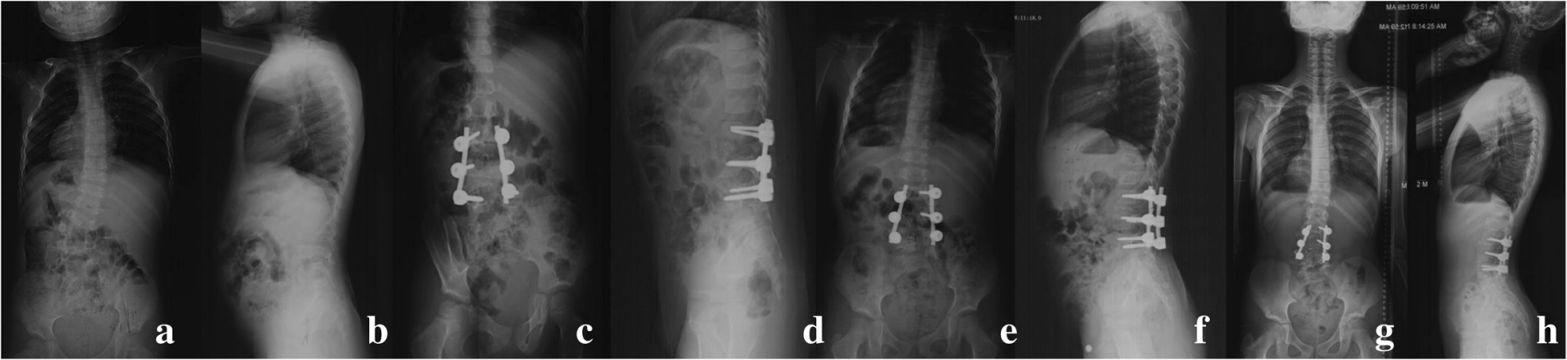
A 3-year-old girl with congenital scoliosis. Pre-operative radiographs (**a**, **b**) showed fully-segmented L3 and L4/5 hemivertebra. Post-operative radiographs (**c**, **d**) showed excellent correction by hemivertebra resection. Six months later, right rod fracture occurred without obvious migration (**e**, **f**). No revision surgery was done and no other rod breakage, rod migration or scoliosis aggravation was observed in 6 years follow-up (**g**, **h**)

**Fig. 3:**
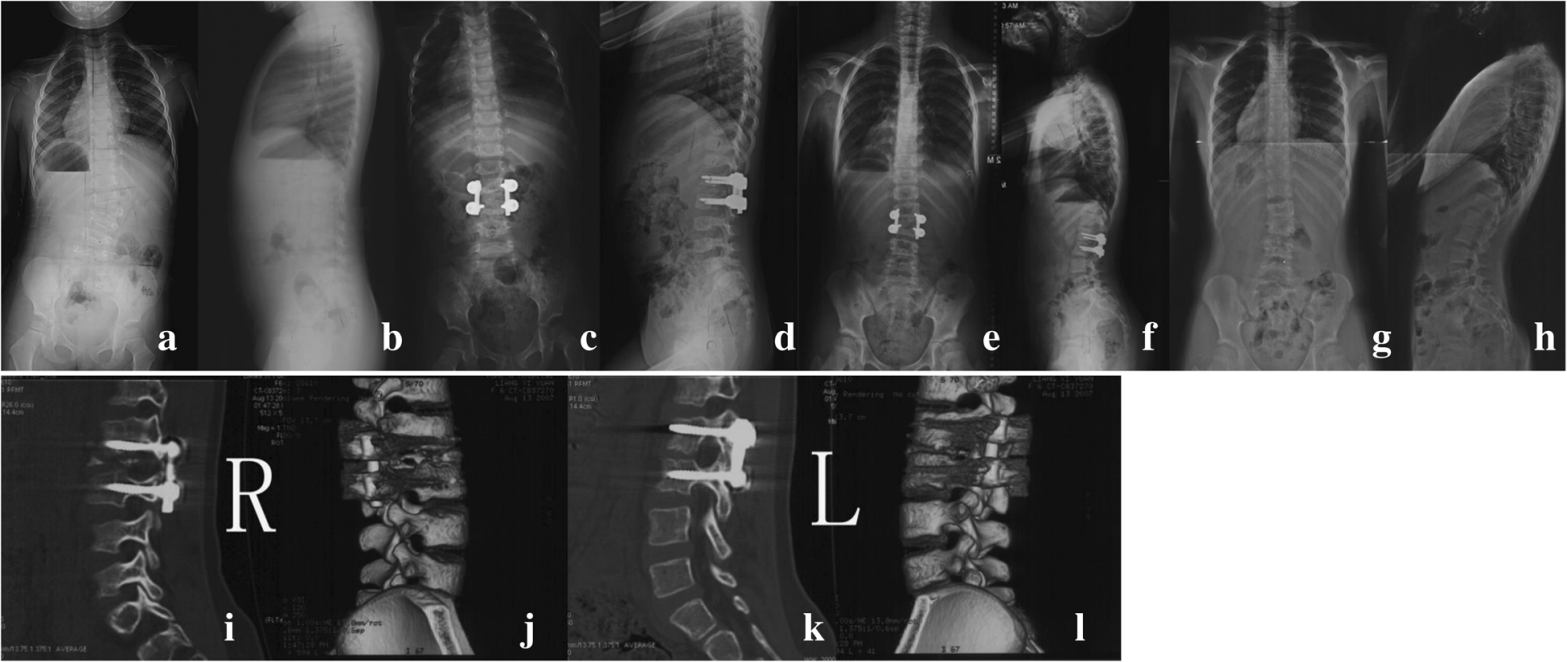
A 3-year-old girl with congenital scoliosis. Pre-operative radiographs (**a**, **b**) showed semi-segmented hemivertebra of L2. Post-operative radiographs (**c**, **d**) showed excellent correction by L2 hemivertebra resection. Radiographs at 7 years follow-up (**e**, **f**) showed L1 pedicles elongation and right pedicle screw dislodgement. CT scan and 3D reconstruction showed solid fusion at the fused segments was achieved and right pedicle screws dislodgement (**i**, **j**, **k**, **l**), and implants was removed (**g**, **h**)

Pedicle misplacements in 2 cases were found postoperatively and revision surgeries were performed (Fig. 1). Only 1 titanium rod breakage occurred 6 months after surgery without loss of correction. No revision surgery was done and no other rod breakage, rod migration or scoliosis aggravation was observed in the follow-up (Fig. 2). 4 implants removal surgeries were performed in four cases for pedicle elongation and dislodging of screw in the follow-up without obvious deformity aggravation (Fig. 3).

A 15 years old patient developed obvious proximal adjacent kyphosis (24°, normal <10°) at the 4-year follow-up after the surgery. Revision surgery was done to correct the adjacent kyphosis. A 3 years old patient developed obvious progressive kyphosis at the 7-year follow-up after thoracic hemivertebra resection with bisegmental fusion. And revision surgery was needed. Wound non-union occurred in 2 cases. No signs of infection were detected. The wound healed well after debridement.

### Risk factors

The association between risk factors and construct/implant related complication were summarized and analyzed in Table 3. Younger age (≤5 years old), lumbar hemivertebra resection or bisegmental fusion appeared to contribute to a higher prevalence of construct/implant related complications, although the number of cases with complications was too small to perform statistical analysis. Sex, age, cage, contralateral bar/rib synostosis, and Cobb angle of segmental scoliosis did not appear to contribute, although this could be due to the small number of cases.

**Table 3:** Association analysis between risk factors and implant-related complications

Risk Factors	Implant-related complications	Non-implant-related complications
	Yes (*n*=7)	No (*n*=109)
Sex		
Male	2 (28.6 %)	60 (55 %)
Female	5 (71.4 %)	49 (45 %)
Age		
≤5 years/old	5 (71.4 %)	29 (26.6 %)
5−10 years/old	1 (14.3 %)	21 (19.3 %)
>10 years/old	1 (14.3 %)	59 (54.1 %)
Location		
lumbar spine	4 (57.1 %)	16 (14.7 %)
others	3 (42.9 %)	93 (85.3 %)
Fusion levels		
bisegmental fusion	5 (71.4 %)	31 (28.4 %)
others	2 (28.6 %)	78 (71.6 %)
Cage		
Yes	0 (0)	15 (11 %)
No	7 (100 %)	94 (89 %)
Contralateral bar/rib synostosis		
Yes	0 (0)	5 (6.1 %)
No	7 (100 %)	104 (93.9 %)
Main curve		
<40°	4 (57.1 %)	37 (33.9 %)
≥40°	3 (42.9 %)	62 (66.1 %)
Correction rate of main curve		
≤80 %	4 (57.1 %)	72 (66.1 %)
>80 %	3 (42.9 %)	37(33.9 %)
Segmental scoliosis		
<30°	3 (42.9 %)	39 (35.8 %)
≥30°	4 (57.1 %)	70 (64.2 %)
Correction Rate of segmental scoliosis		
≤80 %	3 (42.9 %)	43 (39.4 %)
>80 %	4 (57.1 %)	66 (60.6 %)

Statistical analysis between the groups could not be done owing to the low numbers of cases with implant-related complications in this study

In group A (≤5 years old), cases with construct/implant related complications were all female cases with bisegmental fusion and hemivertebra from L1 to S1. In group B or group C, there was only 1 cases with construct/implant related complication. No statistical analysis was done in these groups.

## Discussion

Although posterior hemivertebra resection is a safe and effective technique for congenital scoliosis with hemivertebra, it still has some complications [8, 10, 12, 13, 16–18, 21]. In the early clinical reports of posterior hemivertebra resection, Shono et al. [12] reported a complication rate of 8.33 % in 12 patients (a superficial infection) for a minimum of 2 years follow-up. In Ruf and Harms’ series, 28.5 % of 28 cases with mean age of 3 years and 4 months treated by posterior hemivertebra resection suffered from complications and 62.5 % of complications were due to construct/implant failures [17]. In 2009, they reported a complication rate of 21.5 and 67.9 % of complications were associated with construct/implant failures [11]. In 2011, Zhang reported a 10.8 % complication rate in 56 cases during a mean follow-up of 32.9 months and 65.7 % of complications were associated with construct/implant failures [22]. In our study, the total complication rate was 9.5 and 63.6 % of complications were associated with construct/implant failures. According to earlier reports and our experiences, we found that construct failures still remained the biggest challenge of posterior hemivertebra resection and most of the patients with construct/implant failure were younger than 5 years’ old, with bisegmental fusion or lumbar hemivertebra resection. These risk factors may contribute to a higher prevalence of construct/implant related complications in posterior hemivertebra resection.

Younger age may be one of risk factors for construct/implant related complications for patients with posterior hemivertebra resection. According to previous study, poor bone quality and small size of pedicles are the characteristics of young patients [14]. The bone of young children is very soft and easy to break, and the inner transverse diameters of the pedicles are smaller in very young children than in older ones [23, 24], which may increase the difficulty of implantation. Pedicles may be anomaly or missing in some congenital scoliosis cases, which may contribute to construct/implant related complications.

Bisegmental fusion and lumbar hemivertebra resection may be another risk factors for construct/implant related complications. In order to preserve better mobilization, cases with lumbar hemivertebra were more likely to choose bisegmental fusion. With the limited fused segments, bisegmental fusion would increase the stress on the pedicles, especially on the convex side during the compression. Furthermore, the pullout strength of pedicle screw is low and the screw is easy to loosen or dislodge for poor bone quality and smaller sizes of pedicles in very young children [8, 14]. Besides, some young children have lower compliance with brace after surgery, which may increase the stress on pedicles and cause pedicle fracture.

In our study, 71.4 % cases with construct/implant related complications were younger than 5 years old and 85.7 % chose bisegmental fusion. It is possible that delayed surgery and longer fusion might reduce the construct/implant complications, which would affect the patients’ spinal mobilization and life quality in the future. Besides, delayed surgery will result in structural secondary curve, which need longer fusion [11]. Correction of these rigid curve is more difficult and may increase the risk of neurologic compromise [9, 15]. In order to achieve better correction and preserve more spinal mobilization, we recommend earlier hemivertebra resection and short fusion for most congenital scoliosis cases.

Several strategies are recommended to prevent construct/implant related complications. The first thing is that the hemivertebra must be identified and implants must be carefully prepared during the surgery. Preoperative spine CT scan parallel to pedicle and 3D reconstruction are important for preoperative evaluation and surgical plan and helpful to choose appropriate implants for young cases. Secondly, the whole hemivertebra, including the cartilage, endplates above and below should be completely removed until the bleeding bone to decrease the compression resistant force. Furthermore, contralateral bar or rib synostosis should be resected before compression. Mesh cage is recommended to reconstruct the anterior column and correct the kyphosis to provide postoperative instant stability in cases with large hemivertebra or obvious kyphosis. The last is that tutorization in brace after surgery for at least 3 months. Protection in brace for enough time may be helpful for better fusion results.

This study has several limitations. First, this study was conducted retrospectively and covered a wide range of ages, types and locations of hemivertebra and fusion levels in different cases, and the number of cases with complications was small, which might not draw strong conclusions. Secondly, cases in different ages might have different deformity and flexibility. Although age groups have been used to eliminate these differences, these might still have impact on the final results. Thirdly, most of the cases in this study had not yet reached bone maturity at the last follow-up, and extended follow-up is still needed in the future.

## Conclusion

The occurrence of construct/implant failure related complications in patients with hemivertebra resection is most likely multifactorial. Younger age (≤5 years old), lumbar hemivertebra resection or bisegmental fusion may contribute to a higher prevalence of construct/implant related complications than other cases. However, earlier surgeries and short fusion can preserve more mobile segments and prevent secondary compensatory curve progress, which are more important for congenital scoliosis cases. Measures, such as careful preoperative evaluation and surgical plan with CT scan, sophisticated operation during surgery, usage of cage or cross-links to improve postoperative instant stability, protection in brace and regular follow-up postoperatively, should be taken to reduce construct/implant related complication rate.
